# Asymmetric Dimethylarginine Induced Apoptosis and Dysfunction of Endothelial Progenitor Cells: Role of Endoplasmic Reticulum Stress Pathway

**DOI:** 10.1155/2017/6395601

**Published:** 2017-05-14

**Authors:** Sheng Ye, Xi Zhou, Jiafeng Lin, Peng Chen

**Affiliations:** ^1^Department of Cardiology, The Second Affiliated Hospital and Yuying Children's Hospital of Wenzhou Medical University, Wenzhou 325000, China; ^2^Department of Cardiology, The First Affiliated Hospital of Wenzhou Medical University, Wenzhou 325000, China

## Abstract

Asymmetric dimethylarginine (ADMA), an inhibitor of nitric oxide synthase, is a novel risk factor of cardiovascular disease. Endothelial progenitor cells (EPCs) bear typical endothelial characteristics and are thought to contribute to neovascularization by providing new endothelial cells (ECs) after arterial injury. Many studies have shown that ADMA can induce EPC apoptosis and dysfunction, but the underlying mechanism is not well understood. EPCs from umbilical cord blood were cultured in EGM-2 medium with particular growth factors and supplemented with 10% fetal bovine serum. The cells were treated with different concentrations of ADMA (5, 10, and 50 *μ*mol/L). Endoplasmic reticulum (ER) stress marker levels were examined by western blot analysis. After 24-hour incubation, ADMA induced apoptosis of EPCs and significantly decreased the proliferation, migration, and vasculogenesis capacity of EPCs. We also found that ADMA treatment activated phosphorylated protein kinase RNA-activated-like ER kinase (PERK), a stress sensor protein in the endoplasmic reticulum (ER). The activated PERK induced 78 kDa glucose-regulated protein (GRP-78) and C/EBP homologous protein (CHOP) expression. Additionally, the inhibition of the ER stress pathway by Salubrinal (a specific ER stress inhibitor) can attenuate ADMA-induced apoptosis of EPCs. Overall, these observations indicate that ADMA may induce the apoptosis and dysfunction of EPCs through the ER stress pathway.

## 1. Introduction

Nitric oxide (NO) is a soluble gas continuously synthesized in vascular endothelial cells by nitric oxide synthase (NOS) and plays a crucial role in maintaining normal endothelial function [1]. The bioavailability of this substance depends on the balance between its production and inactivation. Asymmetric dimethylarginine (ADMA) is an endogenous inhibitor of nitric oxide synthase (NOS) enzymes that acts by competition with its substrate L-arginine to reduce nitric oxide production [2, 3]. Impaired nitric oxide production caused by ADMA may facilitate vascular inflammation, thus leading to endothelial dysfunction and eventually atherosclerosis [4]. Many studies suggested that the increased ADMA level in plasma is associated with the risk of major adverse cardiovascular events or death [5, 6]. Thus ADMA is considered an independent risk factor and novel biomarker of cardiovascular events [7, 8].

Endothelial progenitor cells (EPCs) can describe multiple cell types that express a variety of cell surface markers similar to vascular endothelial cells [9]. Emerging evidence showed that EPCs can mobilize from bone marrow stem cell to the peripheral circulation, then proliferate, migrate, and differentiate into mature endothelial cells (ECs) and finally participate in the neovascularization process of injured vessels [10, 11]. Reduced numbers or impaired function of EPCs is associated with atherosclerosis and may lead to cardiovascular disease [12]. It is well known that the mobilization and differentiation of EPCs can be modified by NO and eNOS [13]. Asymmetric dimethylarginine (ADMA), an endogenous NO synthase inhibitor, was reported to inhibit the mobilization, differentiation, and function of EPCs [14]. However, the underlying mechanism is poorly understood.

The endoplasmic reticulum (ER) plays crucial roles in the physiologic regulation of many cellular processes in eukaryotic cells. Transient or prolonged perturbations in ER function and the accumulation of unfolded and misfolded proteins in the ER lead to a condition called ER stress. There are three major ER stress sensors: protein kinase-like ER kinase (PERK), the inositol-requiring enzyme 1 (IRE1), and activating transcription factor 6 (ATF6). These three ER stress sensors are usually bound to the ER chaperone GRP78 and maintained in their inactive forms. However, under ER stress, GRP78 dissociates from these sensors, leading to the activation of the unfolded protein response (UPR) to deal with the accumulated unfolded proteins [15–17]. Prolonged activation of the endoplasmic reticulum (ER) stress pathway can lead to cell dysfunction and death. Several proapoptosis pathways are known to be involved in ER stress-induced cell death. One of the central mechanisms is the PERK-mediated phosphorylation of eukaryotic translation initiation factor 2*α* (eIF2*α*), resulting in translation of the activating transcription factor 4 (ATF4) and the induction of CHOP [18, 19]. A number of studies in cultured endothelial cells and animal models found that ER stress may be involved in endothelial dysfunction and related to vascular pathologies [20–22]. Additionally, a recent study reported that ADMA may induce endothelial cell apoptosis by targeting the PERK-CHOP pathway [23]. However, it is unknown whether ER stress participates in ADMA-induced apoptosis and dysfunction of EPCs. Therefore, in this study we investigated the possible involvement of the ER stress signaling pathway in ADMA-induced apoptosis and dysfunction in EPCs.

## 2. Materials and Methods

### 2.1. Isolation and Cultivation of EPCs

Umbilical cord blood samples (50 mL) were obtained from a fresh umbilical cord of healthy voluntary mothers. The mononuclear cells were isolated using Ficoll gradient centrifugation. After washing with phosphate buffered saline (PBS), 1 × 10^7^ cells were plated in 2 ml of endothelial growth medium-2 (EGM-2; Lonza, Basel, Switzerland) supplemented with 10% fetal bovine serum (FBS, Gibco, Carlsbad, CA, USA) on fibronectin-coated six-well plates and incubated at 37°C in a 5% CO2 incubator. The medium was replaced after the first 24 hours and then changed every 72 hours, and cells that did not adhere were washed away with PBS. The cell growth was observed under an inverted phase contrast microscope. Cells were passaged when the cobblestone-like cells grew to confluence.

### 2.2. Identification and Characterization of EPCs

*CD34 and VIII-Related Antigen Immunohistochemistry.* The second-generation EPCs were fixed with 4% paraformaldehyde for 20 minutes, and then 0.5% H_2_O_2_ in methanol (v/v) was added to inactivate endogenous peroxidase for 10 minutes. After washing with PBS 5 times, the cells were blocked with 10% fetal bovine serum for 20 minutes at 37°C. Next, the cells were incubated with the primary antibodies (CD34 or factor VIII) at 4°C overnight. After three washes with PBS, the cells were treated with the secondary antibody for 30 minutes. According to the standard protocol, the streptavidin-biotin complex was added and incubated for 20 minutes at 37°C. The positive cells were stained brown. Smooth muscle cells served as the negative control.

*Fluorescent Staining*. The adherent EPCs were first incubated with 2.4 *μ*g/ml 1,1′-dioctadecyl-3,3,3′,3′-tetramethylindocarbocyanine-labeled acLDL (DiI-acLDL, Molecular Probe, USA) for 4 hours and then fixed in 4% paraformaldehyde for 10 minutes. Next, the cells were incubated with 10 *μ*g/mL fluorescein isothiocyanate-labeled lectin from ulex europaeus agglutinin-1 (FITC-UEA-1; Sigma USA) for 1 hour at 37°C. After washing with PBS, the slides were observed with an inverted fluorescent microscope (Leica, Wetzlar, Germany).

*EPCs Treatment*. To evaluate whether ADMA can induce apoptosis and dysfunction of EPCs, second-generation EPCs were treated with different concentrations of ADMA (0, 5, 10, and 50 *μ*mol/L) for 24 hours. To determine the relationship between ADMA-induced apoptosis and the ER stress pathway, the EPCs were pretreated with a specific ER stress inhibitor, Salubrinal (25 *μ*mol/L), for 1 hour, followed by treatment with ADMA (50 *μ*mol/L) for 24 hours.

*NO Measurement*. After treatment with different concentrations of ADMA (0, 5, 10, and 50 *μ*mol/L) for 24 hours, the medium was sampled for NO determination using the Griess Reagent method with NO assay kits (Byotime, Haimen, China).

*EPCs Apoptosis Assay*. The cells were fixed with 4% paraformaldehyde. The terminal deoxynucleotidyl transferase-mediated dUTP nick end labeling (TUNEL) assay was applied to detect the cell apoptotic rate in situ according to the manufacturer's protocol (Roche Molecular Biochemicals). TUNEL positive cells were quantified by light microscopy.

*EPCs Proliferation Assay*. The CCK-8 method (Dojindo, Kumamoto, Japan) was used to detect cell proliferation ability [24, 25]. Second-generation EPCs were cultured in a flat-bottomed 96-well plate. After treatment with different concentrations of ADMA (0, 5, 10, and 50 *μ*mol/L) for 24 hours, the cells were incubated with the CCK-8 working solution (10 *μ*L/well) and incubated at 37°C for 2 hours. Finally, cell proliferation ability was determined as the percentage of absorbance as measured using a microplate reader at 450 nm.

*EPCs Migration Assay*. The Boyden chamber assay (Transwell, Costar) was used to evaluate the migratory function of EPCs [26, 27]. First, the adherent EPCs were exposed to ADMA for 24 hours as described above. After detaching with trypsin/EDTA, 2 × 10^4^ cells in serum-free EGM-2 medium were plated in each upper chamber, and EGM-2 medium with 10% FBS was plated in the lower chamber. After 24 hours, the membranes were washed twice with PBS and fixed with 4% paraformaldehyde. The cells attached to the upper side of the membrane were removed using a cotton swab. The membrane was then stained with 0.25% crystal violet and migrated cells were counted with an inverted microscope in 6 random 200x fields.

*EPCs Adhesion Assay*. After exposure to ADMA for 24 hours, the EPCs were harvested and equal amounts were transferred to the wells of a 48-well plate (3 × 10^4^/well). After incubation for 30 minutes at 37°C, nonadherent cells were washed away with PBS, and the adherent cells were counted with an inverted microscope in 10 random 400x fields.

*EPCs Tube Formation Assay*. The vasculogenesis activity of EPCs was assessed by a tube formation assay. Briefly, ECMatrix gel was thawed at 4°C overnight and then transferred at 50 *μ*l/well into a 96-well plate. After incubation at 37°C for 1 hour, the matrix solution became solidified. The experimental EPCs treated with ADMA were harvested and plated (1 × 10^4^ cells/well) on the matrix gel solution with EGM-2 medium and incubated at 37°C for 24 hours. Tube formation was inspected under an inverted light microscope at 200x magnification. The number of formed tubes were counted with an inverted microscope in 6 random fields.

*Western Blot Analysis*. Cultured EPCs were lysed in RIPA lysis buffer with protease and phosphatase cocktail inhibitors for total protein extracts. The protein concentration was determined with the Pierce® BCA protein assay kit (Thermo Fisher, USA). Next, 50 *μ*g cellular protein samples were separated by 8%–12% SDS-polyacrylamide gel electrophoresis and transferred to a PVDF membrane. After blocking with 5% nonfat milk, the membranes were hybridized with antibodies to anti-cleaved caspase-3, anti-p-PERK, anti-PERK (CST, USA), anti-GRP78, anti-CHOP, or anti-GAPDH (Santa Cruz, USA) overnight at 4°C. Horseradish peroxidase-conjugated secondary antibodies were used and then visualized by enhanced chemiluminescence detection reagents. Specific protein expression levels were normalized to the levels of a housekeeping protein. The relative densities were analyzed by Image Quant software.

*Statistical Analysis*. All data were expressed as mean ± SEM. Comparisons between groups were performed by one-way ANOVA. A* P* value less than 0.05 was considered significant. All statistical analyses were performed using the SPSS software 20.0.

## 3. Results

### 3.1. Characterization of EPCs

**Figure 1:**
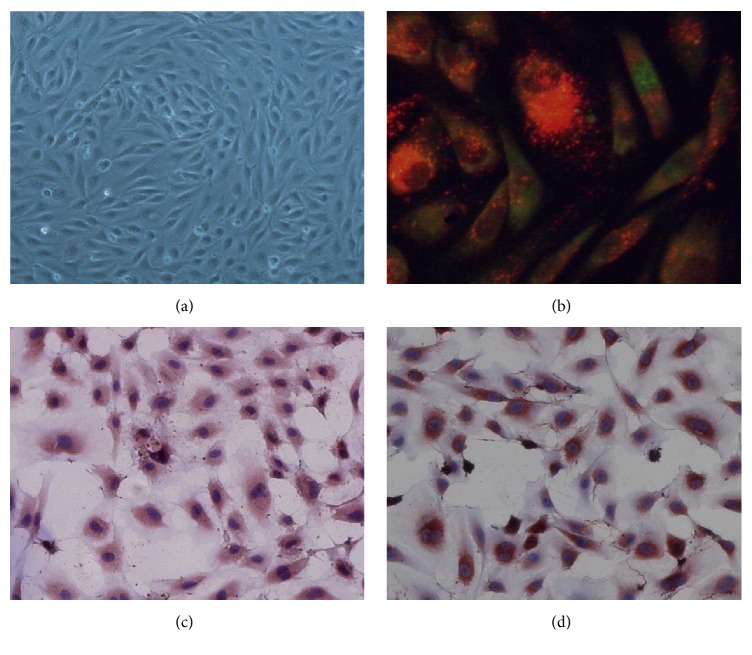
Identification of EPCs culture after 7 days. (a) The cells rapidly reached confluence and showed cobblestone-like morphology highly similar to mature endothelial cells. Images are visualized at 200x magnification. (b) DiI-ac-LDL and FITC-UEA-1 double fluorescent staining at 200x. Double-positive cells are yellow. (c) CD34 immunohistochemistry. Positive cells were stained brown. (d) Factor VIII immunohistochemistry. Positive cells were stained brown.

After being cultivated for 10 days, the adherent cells rapidly reached confluence and showed cobblestone-like morphology that was highly similar to mature endothelial cells (Figure 1(a)). The fluorescent staining was observed using a laser scanning confocal microscope and revealed that the second-generation EPCs were able to uptake DiI-ac-LDL and bind FITC-UEA-1 (Figure 1(b)). Immunohistochemistry staining also showed that EPCs expressed both CD34 and factor VIII (Figures 1(c) and 1(d)). These characteristics were consistent with other studies of EPCs [28].

### 3.2. ADMA Inhibited Nitric Oxide Synthesis of EPCs

**Figure 2:**
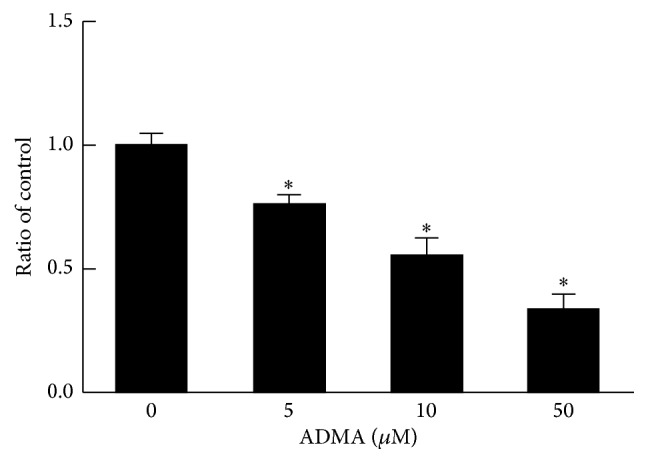
ADMA inhibited nitric oxide synthesis of EPCs. NO assay was applied to determine the effect of ADMA on EPCs NO production. The quantitative graph showed that ADMA (5~50 *μ*mol/L) could significantly inhibit nitric oxide synthesis of EPCs (*n* = 3 per group). Mean ± SEM. ^*∗*^*P* < 0.05 versus control.

The effect of ADMA on EPCs NO production was accessed by Griess Reagent method. Compared with control group, ADMA (5~50 *μ*mol/L) could significantly inhibit nitric oxide synthesis of EPCs (*P* < 0.05) (Figure 2).

### 3.3. ADMA Induces Apoptosis of EPCs

**Figure 3:**
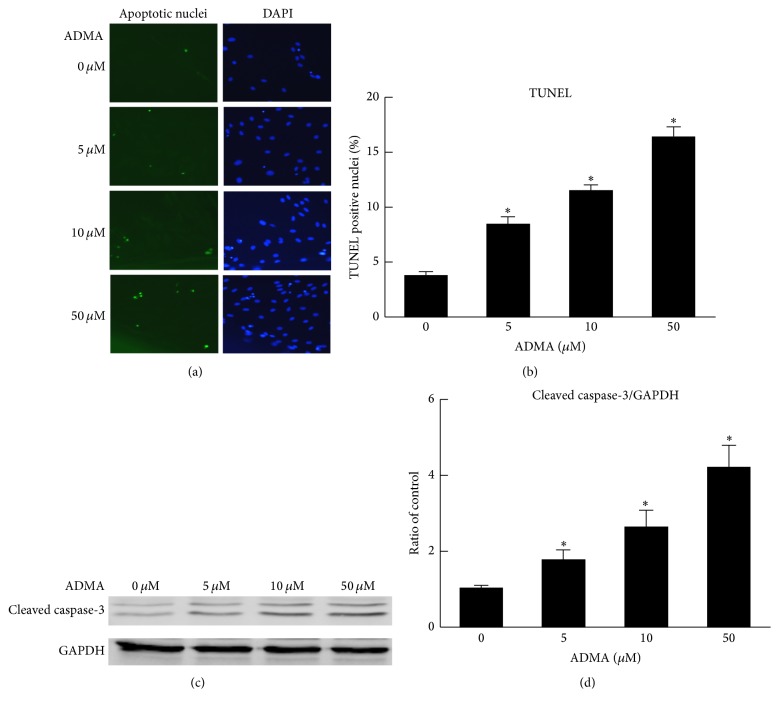
ADMA-induced apoptosis of EPCs. (a and b) The TUNEL assay was applied to determine the rate of ADMA-induced EPCs apoptosis. Apoptotic nuclei were visualized by green fluorescence. DAPI staining (blue) was used as a marker for the cell nucleus. Representative images and the quantitative graph showed that ADMA (5~50 *μ*mol/L) induced EPCs apoptosis in a dose-dependent manner. (c and d) Representative western blots and quantification of cleaved caspase-3. The expression of cleaved caspase-3 induced by AMDA was dose-dependent (*n* = 3 per group). Mean ± SEM. ^*∗*^*P* < 0.05 versus control.

We used the TUNEL staining assay to determine if ADMA induced apoptosis of EPCs. The positive cells were labeled with FITC by green fluorescence and the total cell nuclei were labeled with DAPI by blue fluorescence. As shown in Figures 3(a) and 3(b), after 24-hour incubation, compared with the nontreated control group, various concentrations of ADMA (5~50 *μ*mol/L) induced apoptosis of EPCs in a dose-dependent manner. To further elucidate the role of ADMA-induced apoptosis, we next examined one of the critical apoptosis marker proteins, cleaved caspase-3. Consistent with the TUNEL staining results, the expression of cleaved caspase-3 induced by AMDA also occurred in a dose-dependent manner (Figures 3(c) and 3(d)).

### 3.4. ADMA Induces Dysfunction of EPCs

**Figure 4:**
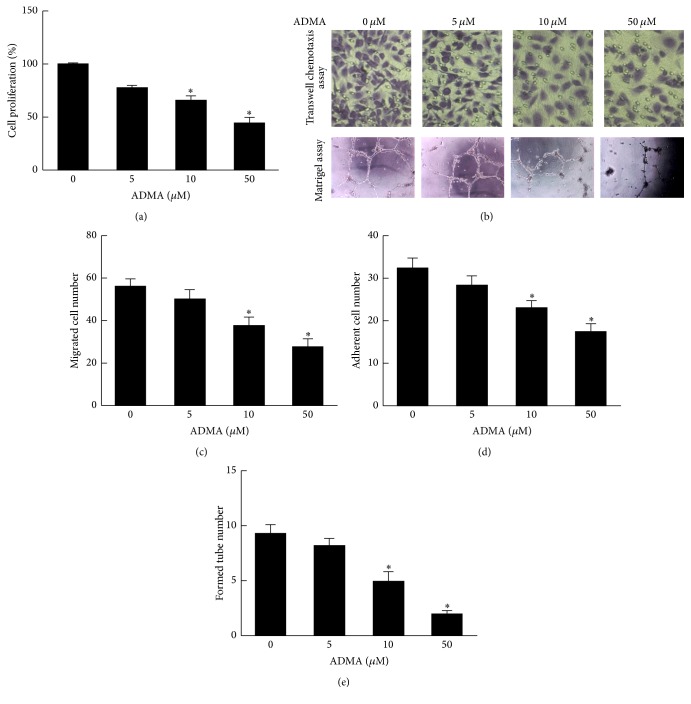
ADMA-induced dysfunction of EPCs. (a) CCK-8 assay was performed to examine the proliferation of EPCs. Compared with the control group, ADMA (5~50 *μ*mol/L) treatment significantly inhibited EPCs proliferation. (b and c) Transwell chemotaxis assay was performed to examine the migration of EPCs. ADMA (10 and 50 *μ*mol/L) treatment inhibited EPCs migration, but 5 *μ*mol/L ADMA did not. (d) ADMA (10 and 50 *μ*mol/L) decreased the number of adherent EPCs. (b and e) ADMA (10 and 50 *μ*mol/L) decreased EPCs tube formation (*n* = 3 per group). Mean ± SEM. ^*∗*^*P* < 0.05 versus control.

To investigate the effects of ADMA on proliferation of EPCs, we performed the CCK-8 assay. Compared with the control group, the proliferation of EPCs was reduced by ADMA (5~50 *μ*mol/L) in a dose-dependent manner (Figure 4(a)).

The effect of ADMA on the migration ability of EPCs was measured with the Boyden chamber assay. Compared with the control group, ADMA (10 and 50 *μ*mol/L) significantly inhibited the migration ability of EPCs (*P* < 0.05), but 5 *μ*mol/L ADMA did not (Figures 4(b) and 4(c)).

Similarly to the migratory function results, the adhesion ability of EPCs was also inhibited by ADMA (10 and 50 *μ*mol/L; Figure 4(d)).

The tube formation ability of EPCs is believed to be very important during neovascularization. After incubation on ECMatrix gel for 24 hours, control EPCs formed an extensive and enclosed tube network. However, this vasculogenesis activity of EPCs was impaired by addition of various concentrations of ADMA (10 and 50 *μ*mol/L; Figures 4(b) and 4(e)). Overall, ADMA causes dysfunction of EPCs.

### 3.5. The Role of ER Stress in ADMA-Induced Apoptosis and Dysfunction of EPCs

**Figure 5:**
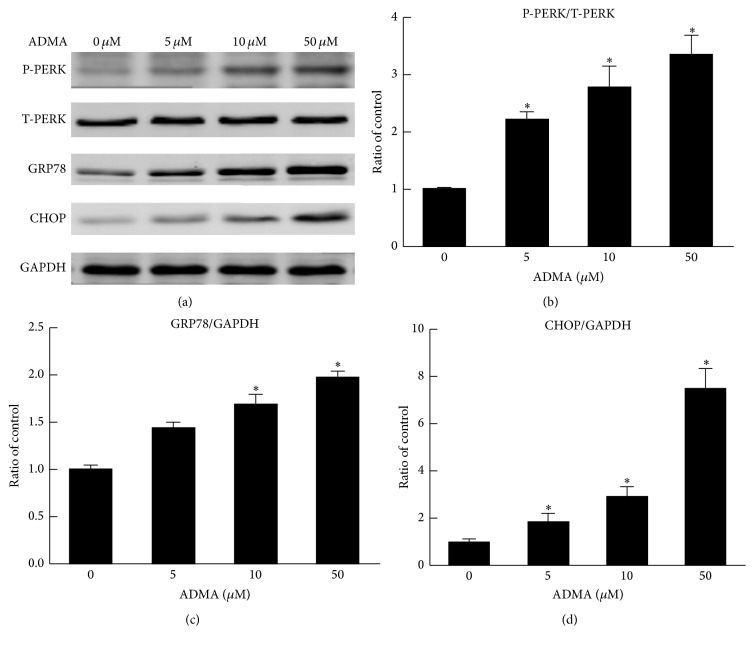
Role of the ER stress pathway in ADMA-induced apoptosis and dysfunction of EPCs. (a and b) Representative western blots and quantification of Phospho-PERK, GRP78, and CHOP. The levels of Phospho-PERK, GRP78, and CHOP were all upregulated after incubation with ADMA (5~50 *μ*mol/L) for 24 h. Mean ± SEM. ^*∗*^*P* < 0.05 versus control.

To investigate whether ER stress involves the ADMA-induced effects on EPCs of apoptosis and dysfunction, we used western blotting to examine the expression of ER stress markers. We found that the expression of Phospho-PERK, GRP78, and CHOP were all upregulated after incubation with ADMA for 24 hours, indicating that ADMA can induce ER stress in EPCs (Figures 5(a)–5(d)).

### 3.6. Inhibition of the ER Stress Pathway Attenuates ADMA-Induced Apoptosis of EPCs 

**Figure 6:**
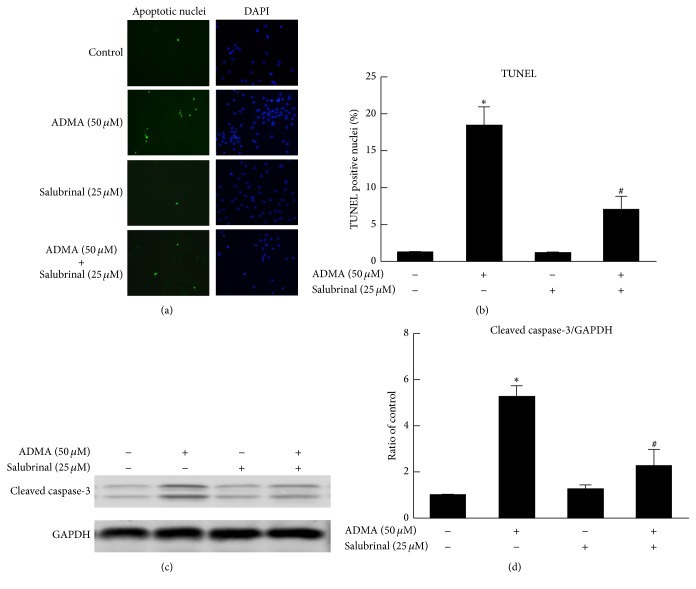
Inhibition of ER stress pathway attenuated ADMA-induced apoptosis of EPCs. The TUNEL assay was applied to determine the apoptosis rate of EPCs. Apoptotic nuclei are visualized by green fluorescence. DAPI staining (blue) is used as a cell nucleus marker. (a and b) Representative images allowed the quantitative analysis of the effect of Salubrinal (a specific ER stress inhibitor) on ADMA-induced apoptosis of EPCs. EPCs were incubated alone or with 50 *μ*M ADMA for 24 hours and with or without 25 *μ*M Salubrinal after 1 hour before treatment. ADMA-induced EPCs apoptosis was attenuated by Salubrinal. (c and d) Representative western blots and quantification of cleaved caspase-3. The expression of cleaved caspase-3 induced by AMDA was also decreased by Salubrinal (*n* = 3 per group). Mean ± SEM. ^*∗*^*P* < 0.05 versus control. ^#^*P* < 0.05 versus ADMA (50 *μ*M).

To determine the relationship of ADMA-induced ER stress and the apoptosis of EPCs, we used Salubrinal, a specific ER stress inhibitor. Our results showed that the inhibition of the ER stress pathway by Salubrinal significantly decreased ADMA-induced apoptosis of EPCs (Figures 6(a) and 6(b)), as well as the level of the apoptosis marker protein cleaved caspase-3 (Figures 6(c) and 6(d)). These results support the model that ADMA causes the apoptotic cell death of EPCs through the ER stress pathway.

## 4. Discussion

In the present study, we showed that ADMA markedly induced EPCs apoptosis and dysfunction in vitro. Furthermore, we identified that ER stress induced by ADMA is involved in EPCs apoptosis and dysfunction.

It is well known that endothelial layers play an important role in maintaining vascular structure. The disruption of endothelial function will initiate atherosclerosis. Recently, many experimental and clinical studies have reported that circulating endothelial progenitor cells (EPCs) can differentiate into mature endothelial cells and then replace the injured endothelium, thereby limiting the development of atherosclerotic processes after vascular injury [10, 29, 30]. Therefore, decreased numbers and function of EPCs will accelerate atherosclerosis and cardiovascular disease. Many traditional coronary heart disease (CHD) risk factors, such as Ox-LDL, advanced age, diabetes, and smoking, were reported to have a detrimental effect on EPCs number and function [30–32].

ADMA, an endogenous inhibitor of nitric oxide synthase (NOS) enzymes, was shown to impair endothelial function and inhibit neoangiogenesis and is considered a novel biomarker of cardiovascular events. The plasma level of ADMA in healthy humans is approximately 1.0 ± 0.1 *μ*mol/l and can increase up to more than 10-fold in patients with coronary artery disease [33]. In our study, we found that treatment of cultured EPCs with various concentrations of ADMA (5~50 *μ*mol/L) induced EPCs apoptosis and dysfunction in a dose-dependent manner, which confirmed the observations of Thum et al. [14], who reported that ADMA plasma levels in patients with coronary artery disease were inversely associated with the number of circulating EPCs and had a strong inhibitory effect on EPCs mobilization, differentiation, and function. This finding suggested that effect of the increased level of ADMA on coronary artery disease may be, at least partly, due to the impairment of EPCs.

Recent studies suggested that ER homeostasis is important in endothelial function [15, 20]. Chronic activation of ER stress is involved in endothelial dysfunction and leads to vascular diseases. ER stress induced by homocysteine can promote EPCs apoptosis in patients with coronary artery disease [22]. Moreover, it was reported that ADMA could induce endothelial cell apoptosis via the ER stress pathway [23]. Hence, we hypothesized that ER stress may be the underlying mechanism of ADMA-induced EPCs apoptosis and dysfunction. We found that ADMA increased phosphorylation of PERK and enhanced the expression of GRP78 and CHOP in a dose-dependent manner.

These results confirmed our hypothesis of the involvement of ER stress in ADMA-induced EPCs apoptosis and dysfunction. In addition, we demonstrated dose-dependent expression of cleaved caspase-3 induced by ADMA. ER stress can activate the caspase cascade during cell apoptosis, including caspase-3 [34]. By investigation of the relationship between ADMA-induced EPCs apoptosis and the ER stress pathway, we found that inhibition of ER stress pathway by Salubrinal (a specific ER stress inhibitor) can attenuate ADMA-induced EPCs apoptosis as well as the expression of cleaved caspase-3. Thus, it is likely that the effect of ADMA in apoptosis of EPCs occurred through the ER stress-mediated activation of caspase-3.

## 5. Conclusions

In conclusion, the results of this study demonstrate for the first time that ADMA induces EPCs apoptosis and dysfunction at least in part through the ER stress pathway and the activation of caspase-3. These findings provide new insights into the mechanism of ADMA-induced EPCs impairment. The modulation of the ER stress signaling pathway may be utilized to develop novel therapeutic strategies for ADMA-associated cardiovascular pathologies.
